# Quercetin and Mesenchymal Stem Cell Metabolism: A Comparative Analysis of Young and Senescent States

**DOI:** 10.3390/molecules29235755

**Published:** 2024-12-05

**Authors:** Alexandra Ivan, Alexandra Teodora Lukinich-Gruia, Iustina-Mirabela Cristea, Maria-Alexandra Pricop, Crenguta Livia Calma, Alina-Georgiana Simina, Călin Adrian Tatu, Atena Galuscan, Virgil Păunescu

**Affiliations:** 1 Department of Functional Sciences, Center of Immuno-Physiology (CIFBIOTEH), University of Medicine and Pharmacy “Victor Babes”, Eftimie Murgu Sq. No. 2, 300041 Timisoara, Romania; ivan.alexandra@umft.ro (A.I.); crenguta.calma@umft.ro (C.L.C.); vpaunescu@umft.ro (V.P.); 2 OncoGen Centre, Clinical County Hospital “Pius Branzeu”, Blvd. Liviu Rebreanu 156, 300723 Timisoara, Romania; mirabela.cristea@oncogen.ro (I.-M.C.); alexandra.pricop@oncogen.ro (M.-A.P.); alina.simina@oncogen.ro (A.-G.S.); 3 Department of Applied Chemistry and Environmental Engineering and Inorganic Compounds, Faculty of Industrial Chemistry, Biotechnology and Environmental Engeneering, Polytechnic University of Timisoara, Vasile Pârvan 6, 300223 Timisoara, Romania; 4 Translational and Experimental Clinical Research Centre in Oral Health, Department of Preventive, Community Dentistry and Oral Health, “Victor Babes” University of Medicine and Pharmacy, 300040 Timisoara, Romania; galuscan.atena@umft.ro; 5 Department I, Department of Preventive, Community Dentistry and Oral Health, “Victor Babes” University of Medicine and Pharmacy, Eftimie Murgu Sq. No. 2, 300041 Timisoara, Romania

**Keywords:** human exfoliated deciduous teeth, fatty acids, mitochondria, senescence, antioxidants

## Abstract

Quercetin is a natural flavonoid renowned for its potent antioxidant, anti-inflammatory, anti-diabetic, and antibacterial properties, making it a highly promising candidate for the treatment of various medical conditions. Our current study investigates the influence of quercetin on energy metabolism, fatty acid composition, oxidative stress gene expression, and sirtuin expression in early- and late-stage passages of stem cells derived from human exfoliated deciduous teeth (SHEDs). Mitochondrial respiration was analyzed by measuring oxygen consumption following a 24 h quercetin treatment, while fatty acid profiles were examined using gas chromatography–mass spectrometry (GC-MS). Additionally, quantitative PCR (qPCR) was used to assess the expression of oxidative stress genes and sirtuins. In younger SHEDs, quercetin enhances metabolic activity and mitochondrial respiration, although higher doses may decrease mitochondrial activity. Conversely, in older, senescent SHEDs, quercetin supports mitochondrial function at lower concentrations but appears to inhibit respiration at higher doses. These results suggest that quercetin may hold therapeutic potential for maintaining SHED viability and function, especially at lower doses in older cells. Further research is essential to fully elucidate a dose-dependent effect of quercetin and optimize its applications in regenerative medicine.

## 1. Introduction

Mesenchymal stem cells (MSCs) are a versatile group of cells found in various organs throughout the body. These cells possess the remarkable ability to self-renew and differentiate into multiple tissue types, maintaining this regenerative potential throughout an individual’s life [1]. Stem cells from human exfoliated deciduous teeth (SHEDs) are stem cells derived from dental pulp tissue of primary teeth during the mixed dentition phase in children. These postnatal stem cells originate from the embryonic neural crest and possess inherent neurotrophic properties, as well as the capacity to differentiate into various cell types [2]. SHEDs offer several biomedical advantages over other MSC types, such as high proliferative potential, minimally invasive harvesting, neuronal differentiation capabilities, neurotrophic capacity, and minimal ethical concerns [3,4]. The therapeutic potential of SHEDs is largely attributed to the paracrine effects of secreted factors, particularly the secretome, with exosomes being a key component. However, despite these advantages, several challenges remain, such as developing a robust GMP-grade manufacturing process, optimizing administration routes, and thoroughly evaluating efficacy and safety in humans, all while ensuring sufficient SHED cell yield and long-term stability [5]. The extensive in vitro expansion of cells is crucial for achieving the necessary cell numbers required for cell-based therapies. However, the regenerative and clinical potential of MSCs declines with prolonged in vitro culture [2,6]. Primary cells can only divide a limited number of times before entering replicative senescence. This process triggers alterations in cell morphology, metabolism, secretory phenotype, and differentiation potential, which can significantly influence experimental results and diminish their therapeutic potential [7,8]. Stem cell exhaustion is also recognized as a hallmark of aging [9,10]. MSCs play a critical role in tissue regeneration, and aging in humans and animals is associated with a reduced number and diminished proliferative potential of MSCs [11]. Accelerated depletion of the MSC pool has been observed in human stem cells and mouse models of premature aging disorders, such as Werner syndrome and Hutchinson–Gilford progeria syndrome [12]. Remarkably, transplantation of mesoderm-derived stem cells from young mice has been shown to extend lifespan and improve fitness in progeroid mice [13,14].

Mitochondria are central to cellular senescence due to their significant role in reactive oxygen species (ROS) production, which contributes to senescence [15]. Senescent cells that lose mitochondrial function often exhibit elevated adenosine triphosphate (ATP) levels, primarily through upregulated glycolysis, indicating that ATP levels alone may not dictate the senescence program [16]. Mitochondrial dysfunction exacerbates cellular aging by increasing oxidative damage and contributing to cellular dysregulation [17]. Quiescent stem cells typically exhibit minimal basal metabolic activity and possess a limited number of mitochondria, primarily relying on glycolysis for energy production [18,19]. Despite their low mitochondrial presence and reduced respiratory activity, these stem cells maintain a functional respiratory chain. The active downregulation of mitochondrial oxidative phosphorylation is crucial for preserving stem cell quiescence and self-renewal, likely serving as a mechanism to minimize ROS production, which acts as a signaling molecule that promotes differentiation. Normally, ROS levels are tightly regulated by cellular antioxidant systems [20]. The sensitivity of stem cells to slight fluctuations in ROS levels makes them responsive to antioxidants [21].

Quercetin, a widely studied flavonoid abundant in fruits, vegetables, and tea, functions as a powerful antioxidant both in vitro and in vivo [22,23]. It is recognized as one of the most extensively researched compounds of its kind, owing to its remarkable pharmacological properties and numerous health benefits. Previous studies have demonstrated quercetin’s diverse pharmacological effects, including its potent oxygen radical scavenging, inhibition of lipid peroxidation, and significant anti-adipogenic, anti-apoptotic, and anti-inflammatory activities, positioning quercetin as a promising senolytic agent [24,25]. Quercetin has been shown to modulate mitochondrial biogenesis, stabilize mitochondrial membrane potential, enhance oxidative respiration and ATP synthesis, regulate redox balance within mitochondria, and influence apoptosis through mitochondria-driven pathways. These findings underscore quercetin’s pivotal role in supporting mitochondrial function, with far-reaching implications for aging, metabolism, and disease prevention [26]. Isocoumarins, structural isomers of certain flavonoid phenolic compounds, are well known for their antioxidant, antimicrobial, and anticancer activities. Notably, 3-aryl isocoumarins share an isomeric relationship with flavonoids such as quercetin [27,28]. Among these, quercetin has garnered particular attention for its significant protective effects against various conditions, including cardiovascular diseases, cancer, diabetes, neurodegenerative disorders, asthma, peptic ulcers, osteoporosis, arthritis, and eye diseases [29].

Our research group previously assessed the biological properties of SHED cells at passages 4 and 10 to investigate changes during extended culture. By passage 10, SHED cells exhibited early signs of senescence, including altered morphology, reduced proliferation, and a slight decline in differentiation potential (Ivan A, 2023 [30]). In this study, we aimed to investigate the effects of quercetin on energy metabolism, fatty acid profiles, oxidative stress gene expression, and sirtuin expression in early- (P5) and late-stage (P16) passages of SHED cells. Mitochondrial respiration was assessed by measuring oxygen consumption after a 24 h treatment with quercetin. Fatty acid composition was analyzed using gas chromatography–mass spectrometry (GC-MS). Additionally, oxidative stress gene expression and sirtuin expression in SHED cells by quantitative polymerase chain reaction (qPCR) were evaluated.

## 2. Results

### 2.1. Senescence-Associated β-Galactosidase (SA-β-Gal) Assay

**Figure 1:**
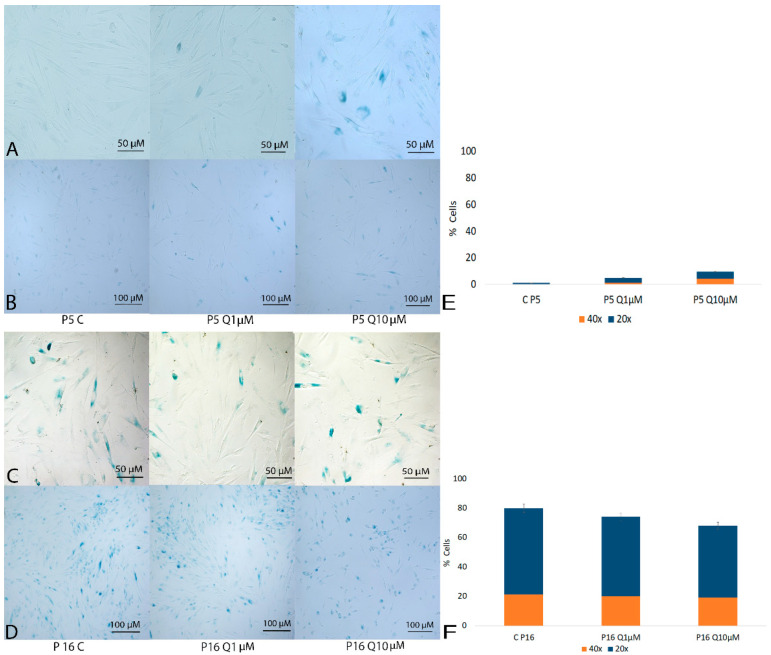
β-galactosidase activity was assessed in SHED cells at early (P5) and late passages (P16). Senescent cells stained blue due to β-galactosidase activity, allowing for visual quantification under a Zeiss Axio Observer Z1 microscope, at magnifications of 200× (**A**,**C**) and 400× (**B**,**D**). Passage 5 (**A**,**B**) represents cells at a younger stage, showing a baseline level of senescence, while passage 16 (**C**,**D**) reflects an advanced stage with likely higher senescence levels. The increase in blue-stained cells at higher passages reflects a greater accumulation of senescent cells as cell passage advances. The percentage of senescent cells was determined by calculating the ratio of β-gal-positive cells to the total cell count, expressed as a percentage for early-passage (**E**) and late-passage (**F**) cells.

When analyzed for senescence-associated β-galactosidase expression, cells at an earlier passage (P5) showed only isolated, weak staining. In the group treated with high quercetin, concentration (10 µM), this expression showed a slight increase, indicating a modest impact of quercetin on cellular senescence markers in younger cells (Figure 1A,B). At late passages, SHEDs transitioned into a senescent phase, as evidenced by increased β-galactosidase (β-gal) staining. This shift was particularly noticeable in the higher number of β-gal-positive cells observed in late passages (P16, Figure 1C,D) compared to early passages. β-galactosidase staining is a well-established marker of cellular senescence, indicating a decline in cellular proliferative capacity and metabolic function. The marked increase in β-galactosidase-positive cells at late passages indicates that SHEDs undergo replicative exhaustion as they reach higher passage numbers, consistent with the behavior of stem cells under prolonged in vitro expansion, which limits their regenerative potential. Quercetin treatment shows limited effectiveness in protecting against the rise in β-gal-positive (senescent) cells at advanced passages, despite an observed reduction in total cell number at higher quercetin concentration (10 µM). The β-gal-positive cell count remains nearly comparable between treated and untreated groups, indicating that quercetin has minimal effect on reducing β-gal-positive cellular senescence markers in aged cells.

### 2.2. Cytotoxicity Assay

**Figure 2:**
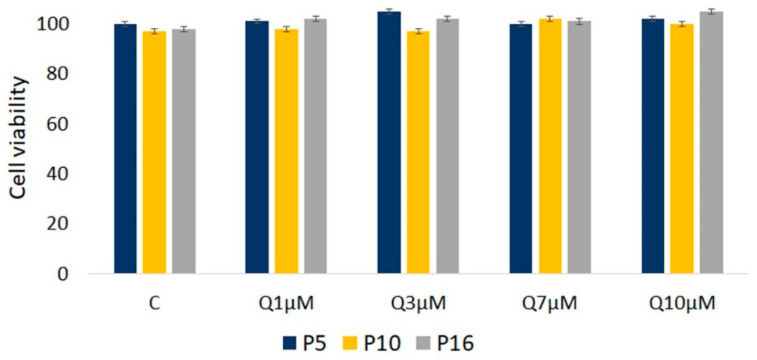
MTT assay. Effect of quercetin on cell proliferation in younger (P5) and older (P16) SHED cell passages across quercetin dose groups (1 µM, 3 µM, 7 µM, and 10 µM) after 24 h of treatment. Data are presented as mean ± SD relative to cells cultured in control media. No significant differences in cell viability were observed.

The MTT ((3-(4,5-dimethylthiazol-2-yl)-2,5-diphenyltetrazolium bromide) assay evaluates the activity of mitochondrial enzymes, primarily NAD(P)H-dependent oxidoreductases, which are active in living cells. These enzymes reduce the yellow MTT compound to insoluble purple formazan crystals, which serve as an indicator of cell viability. The assay compared the proliferation of SHEDs cultured in MEM-α medium supplemented with quercetin at concentrations of 1 µM, 3 µM, 7 µM, and 10 µM for 24 h (Figure 2). No significant differences in cell proliferation were observed between quercetin-treated groups and the control group, indicating the presence of metabolically active cells and suggesting that these quercetin concentrations did not induce cytotoxic effects. No signs of cell death, such as floating cells, were observed, and the cells remained well adhered to the culture surface. These findings confirm that quercetin concentrations of up to 10 µM are non-toxic to SHEDs under the tested conditions.

### 2.3. Oxygen Consumption

The analysis of quercetin’s effect on SHED cells respiration was conducted in live, non-permeabilized cells. Untreated younger cells (P5) displayed high routine respiration (R) (10.66 pmol O_2_/min) and moderate maximum respiration (M) (6.78 pmol O_2_/min), while older untreated cells (P16) showed a decrease in R (6.31 pmol O_2_/min) and leak (L) (1.99 pmol O_2_/min), but an increase in M (15.78 pmol O_2_/min), suggesting that older cells require more energy for maximum respiration but have reduced routine respiration, reflecting aging or senescence.

**Figure 3:**
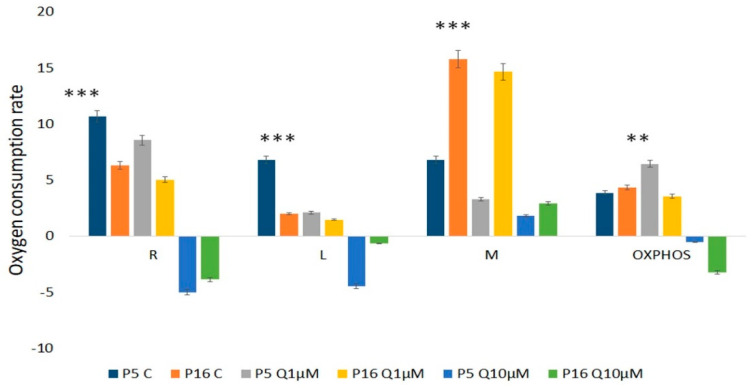
Oxygen consumption rates of SHED cells following quercetin treatment, measured with the Oroboros O2k System. Oxygen consumption was evaluated in early-passage (P5) and late-passage (P16) SHED cells to determine the effects of quercetin treatment at 1 µM and 10 µM. The impact of quercetin on routine respiration (R), maximum respiration (M), leak respiration (L), and oxidative phosphorylation (OXPHOS) is shown. Statistically significant differences between control and quercetin-treated groups are indicated by asterisks ** *p* < 0.001; *** *p* < 0.0001. Representative Oroboros oxygraphs illustrating oxygen consumption and basal respiration, measured using high-resolution respirometry, are available in the Supplementary Files (Figure S1).

In younger cells treated with 1 µM quercetin (P5), R remained high (8.56 pmol O_2_/min), but M decreased (3.29 pmol O_2_/min), indicating reduced maximum respiratory capacity at this low concentration. At the higher concentration of 10 µM, both R and L became negative, indicating significant respiratory inhibition. Older cells treated with 1 µM quercetin (P16) maintained a similar pattern to the control, with a slight reduction in R (5.04 pmol O_2_/min) and a higher M (14.65 pmol O_2_/min), suggesting that 1 µM quercetin may help preserve respiratory efficiency in older cells. However, at 10 µM, both routine respiration and leak were reduced, similar to the effects observed in younger cells, indicating impaired respiration. Overall, the data show that quercetin, especially at 10 µM, reduces oxidative phosphorylation (OXPHOS) in both young and old MSC cells, with a more pronounced effect in older cells. Oxygen consumption rates of SHED cells measured using the Oroboros O2k system in response to quercetin treatment are presented in Figure 3.

In our experiments, quercetin 10 μM led to reductions in both oxygen consumption rate and mitochondrial oxidative phosphorylation, suggesting a dose-dependent inhibition of mitochondrial function. Quercetin was effective at promoting viability, but its effects on mitochondrial function appear to be dose-dependent and passage-specific. Lower concentrations (1 µM) seem to help maintain both viability and mitochondrial function, while higher concentrations (10 µM) can impair respiration despite boosting viability, particularly in younger cells (P5). As cells age (P16), their mitochondrial function declines, but quercetin can help support respiratory capacity at lower concentrations.

### 2.4. Assessment of Fatty Acid Content in SHED Cells

The effect of quercetin on the fatty acid profile in younger and older SHEDs was evaluated by GC-MS. Our results indicate that in early passages of SHEDs, lauric acid levels increase significantly in quercetin-treated groups compared to the control, particularly at the higher quercetin concentration (10 µM). Myristic acid levels also rise in quercetin-treated groups, with the highest concentration (11%) observed in the P5 cells treated with 1 µM quercetin (P5Q1 µM). Palmitic acid remains relatively stable across all groups, averaging over 35%, with a slight increase to 38% in the P16Q10 µM group. Conversely, oleic acid levels display a marked reduction in quercetin-treated groups in both young and older passage cells. Stearic acid levels decrease in the P5Q1 µM group, with a more substantial decline at the higher quercetin concentration (10 µM).

**Figure 4:**
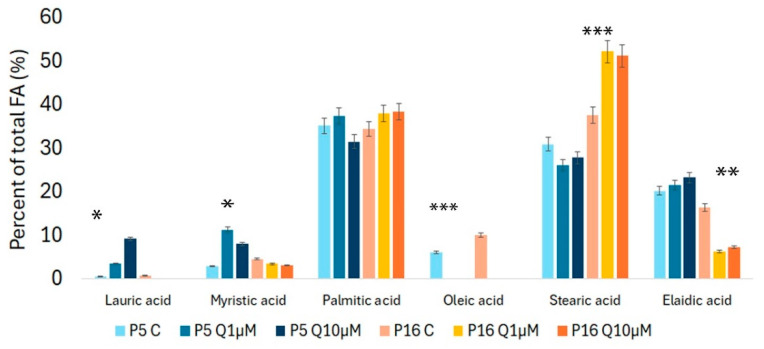
Effect of quercetin on the fatty acid profile in younger and older SHEDs. Data are presented as mean ± SD from three independent experiments. Statistically significant differences between control and quercetin-treated groups are indicated by asterisks * *p* < 0.05; ** *p* < 0.001; *** *p* < 0.0001.

In higher-passage cells (P16), the observed changes are less pronounced than in younger cells for myristic acid. Myristic acid levels decrease slightly in both quercetin-treated groups compared to the control. Palmitic acid remains relatively stable across all groups, with only a minor increase in quercetin-treated cells (both P5Q1 µM and 10 µM), indicating minimal impact by quercetin on this fatty acid. However, quercetin treatment leads to a significant reduction in oleic acid and elaidic acid levels compared to the control. In older cells, stearic acid levels increase significantly in both quercetin-treated concentrations, from 33% to over 50% in quercetin-treated groups. These results are illustrated in Figure 4; also, the representative chromatograms showing the main methylated fatty acids obtained are presented in Figure S2.

**Figure 5:**
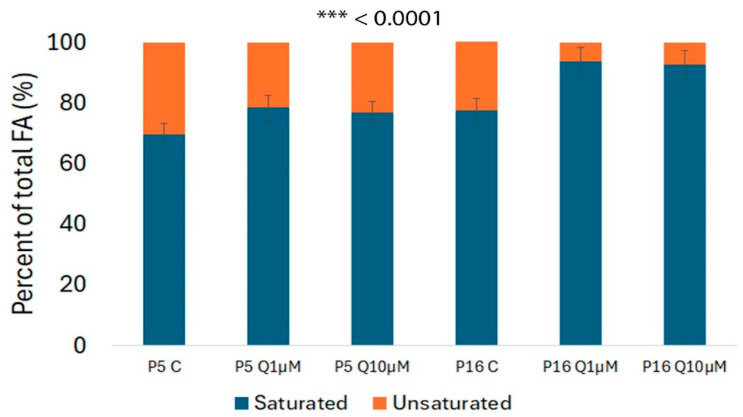
Effect of quercetin (1 µM and 10 µM) on saturated and unsaturated fatty acid levels in younger (P5) and older (P16) SHEDs, as analyzed by GC-MS. Data are presented as mean ± SD from three independent experiments. Statistically significant differences between the older quercetin-treated group and both the younger and older passage control groups are marked with asterisks (*p* < 0.0001).

Regarding the saturated and unsaturated balance, there is a shift towards a higher ratio of saturated fatty acids in the quercetin-treated groups. In the control group of younger cells, the percentage of saturated fatty acids is 69%, which rises to 76% in the group treated with the highest quercetin dose (10 µM). In the P16 groups, saturated fatty acid levels are consistently higher compared to the younger cell groups, exceeding 92% even after quercetin treatment (Figure 5). Although a slight decline in saturated fatty acid levels is observed as the quercetin concentration increases, the overall levels remain significantly elevated.

### 2.5. Antioxidant and Sirtuins Gene Expression Analysis by qPCR

**Figure 6:**
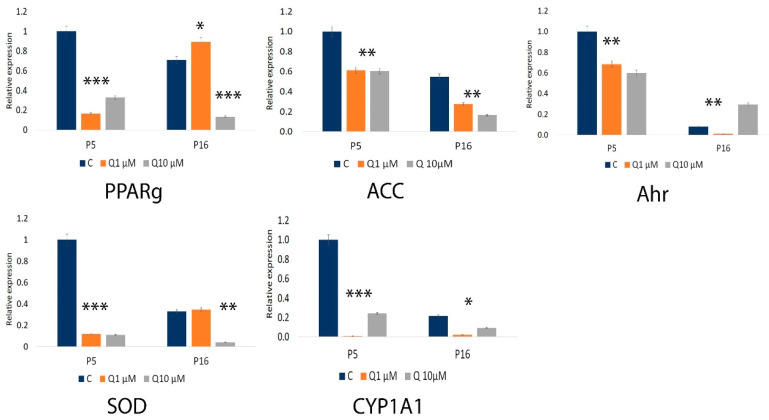
Oxidative stress gene expression levels (PPARγ, ACC, Ahr, SOD, and CYP1A1) in SHEDs were measured by qPCR, with GAPDH used as the reference gene. Data are presented as the mean  ±  SEM from three independent experiments. Statistical significance is indicated as * *p* < 0.05; ** *p* < 0.001; *** *p* < 0.0001.

Quantitative PCR analysis showed a significant decrease in antioxidant gene expression in older cell passages under control conditions. Following quercetin treatment, the effects on antioxidant gene expression differed according to both passage age and dosage. In younger passages, quercetin inhibited gene expression in a dose-dependent manner, reducing the expression of peroxisome proliferator-activated receptor gamma (PPARγ), acetyl-CoA carboxylase (ACC), aryl hydrocarbon receptor (Ahr), superoxide dismutase (SOD), and Cytochrome P450 Family 1 Subfamily A Member 1 (CYP1A1) at both low (1 µM) and high (10 µM) concentrations. Conversely, in older passages, low concentrations of quercetin further reduced Ahr and Cyp1A1 expression (*p* < 0.05), while slightly enhancing the expression of PPARγ (*p* < 0.05) and SOD compared to untreated controls. At 10 µM, quercetin restored Ahr expression to levels exceeding those of untreated controls and partially restored Cyp1A1 (*p* < 0.05) expression, while exhibiting an inhibitory effect on PPARγ (*p* < 0.0001) and SOD (*p* < 0.001). This differential response suggests a modulatory role of quercetin on genes associated with oxidative stress, with effects that are particularly pronounced in senescent or aging cells, potentially pointing to a role in modulating aging-related pathways. In the context of aging, baseline levels of acetyl-CoA were significantly lower in P16 (older) cells compared to P5 (younger) cells, with P5 exhibiting nearly twice the concentration of acetyl-CoA under control conditions. This decline in acetyl-CoA with passage is likely associated with a reduction in both the quantity and oxidative capacity of mitochondria in older cells, reflecting an age-related decrease in fat oxidation. The results are shown in Figure 6; also, the agarose gel electrophoresis of qPCR products for oxidative stress-related genes and sirtuins is presented in Figure S3.

**Figure 7:**
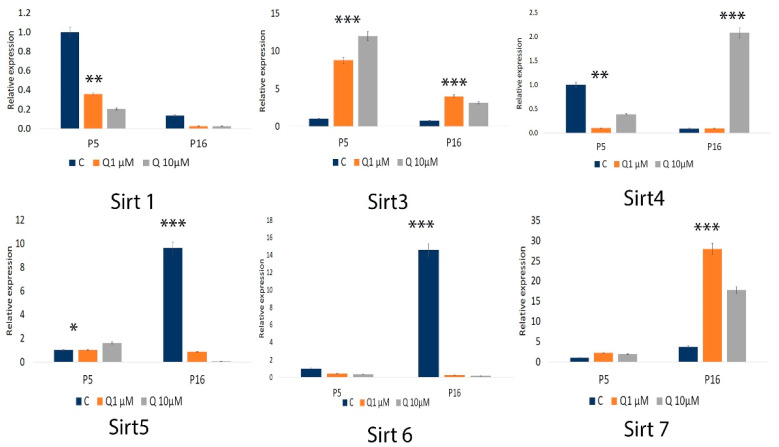
Sirtuins gene expression levels were measured by qPCR, with GAPDH as the reference gene. Data are presented as the mean  ±  SEM from three independent experiments. Statistical significance is indicated as * *p* < 0.05; ** *p* < 0.001; *** *p* < 0.0001.

Quantitative PCR analysis revealed a significant increase in SIRT3 expression (*p* < 0.0001) and SIRT5 (*p* < 0.05) in younger cells following quercetin treatment. A significant reduction in the expression of SIRT1 and SIRT4 genes was observed. In older cell passages, quercetin treatment significantly upregulated SIRT3, SIRT4, and SIRT7 expression (*p* < 0.0001). The effects on antioxidant gene expression varied with both cell passage age and quercetin dosage. In younger cells, quercetin had no significant impact on SIRT6, or SIRT7 expression. In the older control group (without quercetin), a marked increase in SIRT5 and SIRT6 expression was observed compared to younger passages. Figure 7 highlights these results; also, the agarose gel electrophoresis of qPCR products for oxidative stress-related genes and sirtuins is presented in Figure S3.

## 3. Discussion

The present study explores the multifaceted impact of quercetin on stem cell metabolism, focusing on alterations in the mitochondrial respiration, fatty acid profile, oxidative stress-related gene expression, and the modulation of sirtuins. By integrating these key metabolic and regulatory pathways, we aim to clarify the mechanisms underlying quercetin’s influence on stem cell functionality and its potential implications for cellular health and therapeutic applications. To assess quercetin’s impact on mitochondrial activity, we examined its effect on the cellular respiration rate. Prior studies, such as that by Jin et al. (2016), indicated a modest decrease in mitochondrial membrane potential following 8 μM quercetin exposure. At higher doses, however, quercetin may inhibit both respiratory and proliferative functions in aging cells. In our experiments, quercetin was effective at promoting viability, but its effects on mitochondrial function appear to be dose-dependent and passage-specific. These findings indicate a dose-dependent effect of quercetin on mitochondrial function, with potential benefits at lower concentrations and inhibition at higher doses [31].

Aging is closely linked to disruptions in lipid metabolism, as demonstrated by various studies [32]. Changes in lipid profiles may play a crucial role in the cellular and metabolic transformations that characterize aging, impacting membrane integrity, signaling pathways, and inflammatory responses [33]. Quercetin treatment significantly increases the levels of lauric and myristic acids while reducing the unsaturated fatty acids, oleic acids, and elaidic acids, in younger cell passages. This shift suggests that quercetin induces an elevation in saturated fatty acids, which may lead to changes in membrane fluidity or metabolic activity. Lipids containing long-chain and/or saturated fatty acids generally form more rigid, less fluid membranes compared to those composed of shorter and/or unsaturated fatty acids, indicating a potential quercetin-mediated impact on cellular membrane properties and overall cellular function [34]. In older cells, quercetin treatment led to a significant increase in stearic acid levels. This change may represent an adaptive response aimed at counteracting the heightened oxidative stress commonly associated with aging and cellular senescence. Stearic acid has been shown to alleviate oxidative stress by enhancing the activity of endogenous antioxidant enzymes. This protective effect is thought to be primarily mediated through the activation of PPAR-gamma, which triggers the synthesis of protective proteins [35]. By increasing stearic acid (a saturated fatty acid), quercetin reduced the levels of unsaturated fatty acids like oleic and elaidic acids to help maintain membrane stability and prevent oxidative damage.

Lipid breakdown, particularly through mitochondrial β-oxidation, seems to play a paramount role in cellular senescence. Fatty acid oxidation (β-oxidation) is the mitochondrial multi-step process of breaking down a fatty acid into acetyl-CoA units; however, whether this lipid mobilization and breakdown is used for energy production, gene transcription, or generation of SASP (bioactive lipids) is unclear [36]. Several studies suggest that quercetin enhances energy metabolism through a potentially novel mechanism, promoting lipophagy to generate substrates for mitochondrial fatty acid β-oxidation via AMPK signaling activation [37]. This may help explain some of the observed changes in fatty acid profiles following quercetin treatment, including a decrease in lauric acid, myristic acid, oleic acid, stearic acid, and elaidic acid in aged cells. Notably, lauric and myristic acids appear to be preferentially oxidized over palmitic and stearic acids, with lauric and myristic acids serving as primary fuels for respiration in active tissues. Oleic acid, while also an efficient energy source, differs from the other fatty acids by having potential roles in building structural lipids, contributing to cell growth and tissue repair [38,39].

Peroxisome proliferator-activated receptors (PPARs) are critical nuclear transcription factors that play a vital role in lipid metabolism. They regulate processes such as fatty acid oxidation, lipid transport, and lipoprotein assembly by modulating the transcription of target genes involved in these pathways [40,41]. In young cells, our observations indicate that quercetin initially reduces PPARγ expression, reflecting a decreased need for lipid metabolism modulation. This finding is consistent with the research by Wang et al. (2021), which demonstrated that quercetin supplementation significantly lowers PPARγ mRNA expression [42]. Wang et al. (2022) suggest that quercetin may influence the AMPK/PPAR signaling pathway, thereby impacting lipid metabolism [43]. In older cells, low doses of quercetin may modestly activate PPARγ, bringing its levels close to those of untreated young controls. However, at higher quercetin doses, oxidative stress is exacerbated, leading to a significant suppression of PPARγ as cells conserve resources. The effects of quercetin on PPARγ expression vary with passage, as older cells exhibit a more pronounced inhibition of PPARγ at elevated doses, likely due to their increased susceptibility to mitochondrial stress.

In the context of aging, baseline levels of acetyl-CoA were significantly lower in older cells compared to younger cells, which exhibited nearly twice the concentration of acetyl-CoA under control conditions. This decline in acetyl-CoA with passage is likely associated with a reduction in both the quantity and oxidative capacity of mitochondria in older cells [44]. Treatment with quercetin further decreases acetyl-CoA levels in both P5 and P16 cells relative to their controls. Notably, this reduction is more pronounced in the older P16 cells, where acetyl-CoA levels drop by approximately 50%, compared to a 39% reduction observed in P5 cells. These findings suggest that quercetin significantly impacts acetyl-CoA levels, with older cells showing greater sensitivity to its effects. This may indicate that quercetin influences mitochondrial metabolism in an age-dependent manner, potentially through mechanisms related to fatty acid oxidation and mitochondrial respiratory function.

Röhrdanz et al. (2003) reported that quercetin, when administered at concentrations ranging from 5 to 100 µM, significantly reduced the mRNA expression of key antioxidant enzymes in rat hepatoma H4IIE cells, specifically manganese superoxide dismutase (MnSOD), glutathione peroxidase (GPx), and copper–zinc superoxide dismutase (CuZnSOD) by 30–40% [45]. In our experiments, quercetin antioxidant properties appeared to reduce the need for SOD in young cells with lower baseline oxidative stress. However, at higher doses, its pro-oxidant effects become significant, particularly in older cells with impaired mitochondrial function. Gao et al. (2018) highlighted that high quercetin concentrations disrupt glutathione (GSH) homeostasis, potentially increasing oxidative stress. This stress may downregulate SOD activity, especially in older cells with limited capacity to adapt their antioxidant defenses. While low doses of quercetin may offer protection, higher doses can overwhelm the antioxidant system, exacerbating oxidative damage and further reducing SOD activity [46].

Dietary flavonoids, which are abundant in tea, fruits, wine, and vegetables, are particularly effective ligands for AhR, with quercetin being especially noteworthy [47]. Many dietary-derived ligands, however, are considered weak AhR agonists, activating the receptor at low concentrations while exhibiting antagonistic effects at higher concentrations [48]. Initially identified as an environmental sensor, AhR is now recognized as a key regulator of biological rhythms and oxidative stress, playing a crucial role in development and aging by supporting cellular health and adaptability [49]. In our study, we observed a decline in Ahr expression in senescent (P16) cells, consistent with the aging phenotype. However, quercetin treatment, particularly at 10 µM, significantly increased Ahr expression in these older cells, suggesting its role in restoring AhR-associated pathways linked to detoxification, metabolism, and senescence. This dose-dependent recovery may indicate that quercetin mitigates aspects of cellular aging through AhR modulation. In young cells (P5), baseline Ahr activity remained high, supporting normal cellular functions and low stress levels. Quercetin appeared to downregulate Ahr expression, potentially as a protective mechanism to avoid unnecessary activation of xenobiotic and inflammatory pathways. This response aligns with quercetin’s dual role: acting as an antioxidant at low doses and as a pro-oxidant or signaling modulator at higher doses [50]. At low doses, quercetin inhibited Ahr and reduced Cyp1A1 expression, reflecting a downregulation of xenobiotic pathways in the absence of significant stress. In contrast, higher doses induced mild stress, slightly increasing Ahr and Cyp1A1 expression, as the cells activated detoxification pathways to adapt to quercetin’s effects. These findings highlight quercetin’s context-dependent influence on AhR activity, particularly in aging cells where oxidative stress and mitochondrial dysfunction are more pronounced [50].

Sirtuins are a family of NAD+-dependent deacetylases that play vital roles in regulating cellular metabolism, stress responses, and the aging process [51]. Recent research by Yoshida et al. (2024) demonstrated that quercetin enhances the expression of SIRT1, SIRT3, and SIRT6 during trophoblast syncytialization, contributing to improved mitochondrial function and reduced oxidative stress during this process. In our study, quercetin influenced sirtuin expression differently in young and older cells [52]. In younger cells (P5), quercetin reduced SIRT 1 and increased SIRT3 expression, highlighting its essential role in maintaining mitochondrial health. SIRT1 is an NAD+-dependent protein deacetylase that is known to halt the cell cycle and induce DNA repair, slow down apoptosis, and regulate premature cell senescence, while SIRT3 is crucial for regulating mitochondrial oxidative stress and bioenergetics by promoting the expression of genes involved in mitochondrial DNA repair via AMPK signaling [53,54,55]. Notably, quercetin did not affect SIRT5 or SIRT6 in younger cells. In older cells, quercetin significantly upregulated SIRT3, SIRT4, and SIRT7, potentially enhancing survival pathways to counteract age-related stress. In contrast, older control cells exhibited elevated levels of SIRT5 and SIRT6, likely as compensatory responses to age-related stress. SIRT5 and SIRT6 are both implicated in maintaining mitochondrial function and ensuring genomic stability [56,57]. Following quercetin treatment, SIRT5 and SIRT6 levels were restored to those observed in young control cells. SIRT6 is particularly important for protecting and repairing cellular damage caused by oxidative stress; recent studies have shown that SIRT6 helps safeguard cells from DNA damage associated with oxidative stress, suggesting its role in regulating redox-related cellular homeostasis [58]. This function is crucial for preserving the viability of stem cells, as SIRT6 may act as a positive modulator of the NRF2-HO-1 antioxidant pathway in human mesenchymal stem cells (hMSCs) [12,58]. Furthermore, SIRT7, which is associated with ribosomal function and genomic stability [59], increased in response to quercetin treatment, particularly at lower doses, indicating an adaptive stress response in older cells. This upregulation of SIRT7 might serve as a protective mechanism that enhances cellular resilience [17]. Additionally, studies have shown that SIRT7 is rapidly and transiently recruited to DNA damage sites in a PARP1-dependent manner, playing a critical role in the efficient repair of double-strand breaks [60]. These findings highlight the nuanced roles of SIRT7 and quercetin in modulating cellular responses to stress and aging. The observed upregulation of SIRT7 at lower doses of quercetin suggests a potential therapeutic window for enhancing genomic stability and stress resilience in aged cells.

This study highlights the importance of dose optimization in considering quercetin for anti-aging or regenerative therapies. It underscores the strengths of evaluating the biological properties of quercetin, one of the most abundant plant-derived flavonoids. The carefully selected analysis methods proved efficient and reliable, enabling a cost-effective assessment of quercetin’s effects on SHEDs and providing valuable insights into critical cellular metabolic processes. However, the study is not without limitations. While the methodologies were robust, complementary techniques such as Western blotting, immunofluorescence staining, ELISA, and flow cytometry could offer additional validation, at the protein level of specific gene expression and activity.

While current studies primarily focus on the short-term effects of quercetin (up to 24 h), there is a significant gap in research regarding its long-term impact on stem cells. Exploring sustained effects is essential, as it could provide crucial insights into quercetin’s potential for regenerative therapies and its role in cellular aging. Nevertheless, the long-term effects, which are critical for evaluating quercetin’s therapeutic potential, were beyond the scope of this study, and should also be examined in in vivo experiments. Moreover, the reliance on an in vitro model, although effective for understanding cellular mechanisms, cannot fully replicate the complexity of in vivo systems, including systemic interactions, tissue-specific architecture, and the broader physiological context. This limitation restricts the direct applicability of these findings to therapeutic scenarios, although the primary cell type used in our study is a suitable proxy for investigating the pharmacological effects of quercetin, as these cells are of human origin and their metabolic pathways are unaltered compared to those of some cell lines. Nevertheless, in order to address these challenges and acquire a more accurate picture, future research should incorporate animal studies to bridge the gap between cellular findings and clinical applications. Exploring quercetin’s bioavailability, multifaceted biological actions, and involvement in diverse metabolic pathways is essential. Additionally, investigating the mechanistic interplay between stem cell metabolism, quercetin treatment, and age-related cellular changes could open new avenues for targeted interventions in age-associated pathologies.

Our findings suggest that quercetin, through its potent antioxidant and metabolic regulatory properties, holds promise for regenerative medicine by enhancing stem cell function and mitigating age-related cellular decline. These properties may have significant implications for addressing age-related diseases. However, translating these insights into clinical applications will require further investigation into quercetin’s bioavailability, optimal dosing strategies, and long-term safety, paving the way for its therapeutic potential in age-associated conditions. Future studies are warranted to further elucidate the mechanistic interplay between stem cell metabolism, quercetin treatment, and age-associated cellular changes, paving the way for targeted interventions in age-related pathologies.

## 4. Materials and Methods

### 4.1. Cell Culture and Quercetin Treatment

Deciduous teeth were collected from children aged between 7 and 12 years after obtaining informed parental consent, following ethical standards, including the 1964 Helsinki Declaration. After cleaning the tooth surfaces, the dental pulp tissue was extracted, rinsed in phosphate-buffered saline (PBS) (Gibco, Life Technologies, Bleiswijk, The Netherlands), and cut into 0.5–1 mm^3^ fragments. These fragments were placed in 6-well plates containing a supplemented culture medium, and SHED were isolated from the tissue explants.

The cells were cultured in Minimum Essential Medium (MEM) alpha (Gibco, Life Technologies, Paisley, UK), supplemented with 10% fetal calf serum (FCS); (PromoCell, Heidelberg, Germany) and 1% penicillin–streptomycin (Gibco, Life Technologies, Grand Island, NY, USA). The culture medium was refreshed twice per week, and cells were passaged upon reaching 90% confluence. Quercetin (Sigma-Aldrich, St. Louis, MO, USA) was initially dissolved in dimethyl sulfoxide (DMSO; Sigma-Aldrich, Ayrshire, UK) to prepare a 10 mM stock solution, which was further diluted in the culture medium to final concentrations of 1, 5, 7, and 10 µM. The cells were then incubated with quercetin for an additional 24 h.

### 4.2. Senescence-Associated β-Galactosidase (SA-β-Gal) Assay

Cellular senescence was assessed using the SA-β-gal staining method, utilizing the Senescence Cells Histochemical Staining Kit (Sigma-Aldrich; St. Louis, MO, USA). Cells were seeded in 8-well plates at a density of 2 × 10⁵ cells per well and cultured in MEM alpha supplemented with 10% FCS and 1% penicillin–streptomycin. After overnight incubation, cells were treated 1 µM and 10 µM with quercetin for 24 h.

Staining was conducted 24 h post-treatment according to the manufacturer’s instructions. Cells were incubated overnight at 37 °C in a CO_2_;-free environment to facilitate staining development. Senescence was quantified by counting blue-stained cells, indicative of SA-β-gal activity, in five distinct fields under a phase-contrast microscope (Zeiss, Axio Observer Z1, Jena, Germany) at 200× and 400× magnifications. The percentage of senescent cells (blue-stained) was calculated as the ratio of β-gal-positive cells to the total cell count, expressed as a percentage: (%) as 100 × (β-gal-positive cells/total number of cells).

### 4.3. Cytotoxicity Assay

To evaluate the effect of quercetin on cell proliferation, an MTT assay was conducted. Briefly, SHED cells were seeded in 96-well plates at a density of 7 × 10^3^ cells/well and cultured in complete medium for 24 h. Following incubation, cells were treated with quercetin at various concentrations (0, 1, 5, 7, and 10 µM) and cultured for an additional 24 h. The rationale for selecting quercetin doses in the range of 1–10 µM was based on their frequent use in the literature as effective low to medium concentrations for modulating stem cell metabolism, while also allowing flexibility for future studies exploring long-term effects, without triggering some toxic effects. Cell cytotoxicity was assessed by MTT assay (3-(4,5-dimethylthiazol-2-yl)-2,5-diphenyltetrazolium bromide), according to the protocol provided with the MTT Cell Proliferation Assay Kit (Abcam, Cambridge, UK). After the incubation period, 10 µL of MTT reagent was added to each well and incubated for 3 h at 37 °C. The MTT-containing medium was then removed, and 100 µL of MTT solvent was added to each well to dissolve the formazan crystals formed by viable cells. The absorbance was measured to evaluate cell viability. The absorbance, proportional to the number of viable cells, was measured at 590 nm with a reference at 655 nm using a Tecan microplate reader (spectrophotometer Tecan i-control, 1.10.4.0 infinite 200Pro). Cell viability (%) was calculated as 100 × (absorbance of treated cells/absorbance of control cells).

% Inhibition = (100 × (control cells-sample cells))

### 4.4. Oxygen Consumption/Mitochondrial Respiration Oroboros

To assess oxygen consumption, trypsinized cells were resuspended in culture medium and introduced into the Oxygraph-2K system (Oroboros Instruments, Innsbruck, Austria) chamber for analysis. After sealing the chambers, routine respiration was recorded. Oligomycin (2.5 μM) (Sigma-Aldrich, St. Louis, MO, USA) was added to inhibit ATP synthase, enabling measurement of non-ATP-linked respiration. To evaluate the non-phosphorylating capacity of the electron transfer system (ETS), carbonyl cyanide 4-(trifluoromethoxy) phenylhydrazone (FCCP) (Sigma-Aldrich, St. Louis, MO, USA) was titrated in stepwise increments (0.5 μM) to uncouple the mitochondria, stimulating maximum respiratory capacity. Respiration was subsequently inhibited through sequential additions of Rotenone (0.5 μM) (Sigma-Aldrich, St. Louis, MO, USA) and Antimycin A (2.5 μM) (Sigma-Aldrich, St. Louis, MO, USA) to block complex I and complex III, respectively. Prior to measurements, cells were pre-incubated with 1 µM and 10 µM quercetin for 24 h.

### 4.5. Assessment of Fatty Acid Content in SHED Cells

To analyze the fatty acid content, 10^6^ SHED cells per experimental condition were used. Cells were washed multiple times with ice-cold PBS and then centrifuged at 1500 rpm for 10 min to obtain a pellet for lipid extraction. Lipids were extracted by resuspending the cell pellet in 400 µL of a chloroform/methanol solution (2:1 ratio), followed by centrifugation at 4000 rpm for 5 min. The chloroform layer, containing lipids, was transferred to a clean tube, then mixed with 200 µL of water and 100 µL of chloroform, vortexed, and centrifuged again at 4000 rpm for 10 min. The chloroform layer was collected and evaporated under nitrogen to dryness. The dried residue was saponified by adding a 0.45 M natrium hydroxide solution and heated at 65 °C for 1 h, and then neutralized with 0.45 M HCl. The mixture was extracted two times with 2% acetic acid in hexane, and the organic layer was dried under nitrogen. The residue was derivatized with 100 µL acetyl chloride in 5 mL methanol for 45 min. The reaction was stopped with 3 mL of 0.25 M potassium carbonate, and the resulting fatty acids methyl esters were extracted in 1 mL hexane for 1 h. The methylated fatty acids were analyzed using GC-MS.

All solvents and reagents used for extraction, derivatization, and GC-MS were GC-grade, obtained from Sigma-Aldrich (St. Louis, MO, USA). Analyses were conducted on an Agilent 6890 gas chromatograph coupled with a 5973 MSD quadrupole mass spectrometer (Agilent Technologies, Santa Clara, CA, USA), with separations on a DB-WAX capillary column (30 m × 0.25 mm × 0.25 µm) at a helium flow rate of 1 mL/min. The column oven temperature was ramped at 6 °C/min from 50 °C to 250 °C. Samples were injected in splitless mode with an inlet temperature of 230 °C, and mass spectrometer source was set at 150 °C.

Compounds were monitored in full-scan mode (m/z 50–600 Da) with ionization energy set to 70 eV. Compounds were identified after a solvent delay of 4 min by comparing spectra to the NIST11 mass spectral library using ChemStation software, version B.01.00 (Agilent Technologies, Palo Alto, CA, USA). To ensure accuracy and eliminate contamination risks, solvent blanks and control samples were analyzed under identical GC-MS conditions.

### 4.6. Antioxidant and Sirtuins Gene Expression by qPCR

Total RNA was isolated from both control and experimental groups using the established Trizol method (Invitrogen, Carlsbad, CA, USA). The concentration of RNA was accurately quantified with a Nanodrop ND-1000 spectrophotometer, and its purity was assessed through 260/280 absorbance ratios, which ranged from 1.9 to 2.02, confirming the high quality of the RNA samples.

For cDNA synthesis, 1 μg of total RNA was reverse-transcribed in a 20 μL reaction using the RevertAid First Strand cDNA Kit (Thermo Scientific, EU, Lithuania). Quantitative real-time PCR was subsequently conducted on the synthesized cDNA using the KiCqStart SYBR Green qPCR ReadyMix (Sigma, St. Louis, MO, USA) on a LightCycler 480II Roche instrument. We targeted key genes involved in the oxidative stress response, including PPARγ, Acetyl-CoA, SOD, Cyp1A1, and Ahr, to gain insights into the molecular mechanisms underlying the antioxidant enzyme response. Additionally, the expression of sirtuins 1–7 was analyzed to further explore their potential roles in promoting cell survival and longevity. Primer sequence is included in Supplementary Files (Table S1).

### 4.7. Statistical Analyses

To evaluate differences between the experimental groups, one-way analysis of variance (ANOVA) was conducted. The ANOVA was used to determine whether significant differences existed among the three experimental groups. This was followed by pairwise post hoc *t*-tests to identify specific group differences (between two groups). All statistical analyses were performed using Microsoft Excel, Office Pro Plus, 2021. Parametric data are presented as mean ± standard deviation (SD). Statistical significance was determined at a *p*-value threshold of less than 0.05, with results categorized as follows: * *p* < 0.05, ** *p* < 0.001, *** *p* < 0.0001, representing increasing levels of statistical confidence (significant, very significant, and very strong statistical significance).

## 5. Conclusions

Quercetin exerts a multifaceted and passage-dependent influence on stem cells from human exfoliated deciduous teeth, affecting cellular viability, mitochondrial function, fatty acid composition, and the expression of oxidative stress genes and sirtuins. In younger SHEDs, quercetin enhances metabolic activity and mitochondrial respiration, although higher concentrations (10 µM) may negatively impact mitochondrial efficiency. Conversely, in older, senescent cells (passage 16), quercetin supports mitochondrial function at lower concentrations (1µM) but appears to inhibit respiration at elevated doses (10 µM). The effects of quercetin on fatty acid metabolism are particularly pronounced in younger cells (passage 5), where it increases the levels of lauric and myristic acids, while decreasing oleic acid levels. Additionally, quercetin’s modulation of oxidative stress gene expression and sirtuin levels in older cells (passage 16) suggests its potential to counteract some of the changes associated with cellular senescence. Overall, quercetin may offer therapeutic potential for maintaining the viability and functionality of SHEDs, especially at lower concentrations in older passages.

In this study, we focused on the short-term effects of quercetin treatment to establish a foundational understanding of its immediate impact on stem cell metabolism and viability. While the long-term effects were beyond the scope of this work, their importance for evaluating quercetin’s therapeutic potential is recognized. Future research will explore the prolonged influence of quercetin on differentiation capacity, epigenetic stability, and overall stemness in both young and senescent cells, aiming to elucidate its complex dose-dependent effects and optimize its application in regenerative medicine.

## Data Availability

Data are contained within the article and Supplementary Materials.

## Supplementary Materials

The following supporting information can be downloaded at: https://www.mdpi.com/article/10.3390/molecules29235755/s1, Figure S1. Coupled and uncoupled respiration of intact cells: representative oroboros oxygraphs depicting oxygen consumption of SHED cells showing basal oxygen consumption, measured using high-resolution respirometry. Traces include oligomycin-insensitive respiration (following oligomycin addition) and subsequent responses to sequential additions of FCCP (to assess maximal respiratory capacity), rotenone (Rot), and antimycin A (AmyA) to inhibit specific mitochondrial complexes; Figure S2. Representative chromatogram detected by GC-MS showing major fatty acids methyl esters (FAME): lauric acid methyl ester (12:0), myristic acid methyl ester (14:0), palmitic acid methyl ester (16:0), stearic acid methyl ester (18:0), oleic acid methyl ester (18:1 cis 9), and elaidic acid methyl ester (18:1 trans 9); Table S1. Primer sequence for qPCR analysis; Figure S3. Agarose gel electrophoresis of qPCR products for oxidative stress-related genes and sirtuins.

## References

[1] Blanc K.L., Pittenger M.F. (2005). Mesenchymal Stem Cells: Progress toward Promise. Cytotherapy.

[2] Wang H., Zhong Q., Yang T., Qi Y., Fu M., Yang X., Qiao L., Ling Q., Liu S., Zhao Y. (2018). Comparative Characterization of SHED and DPSCs during Extended Cultivation In Vitro. Mol. Med. Rep..

[3] Kashyap R. (2015). SHED-Basic Structure for Stem Cell Research. J. Clin. Diagn. Res. JCDR.

[4] Brar G.S., Toor R.S.S. (2012). Dental Stem Cells: Dentinogenic, Osteogenic, and Neurogenic Differentiation and Its Clinical Cell Based Therapies. Indian J. Dent. Res..

[5] Anoop M., Datta I. (2021). Stem Cells Derived from Human Exfoliated Deciduous Teeth (SHED) in Neuronal Disorders: A Review. Curr. Stem Cell Res. Ther..

[6] Miura M., Gronthos S., Zhao M., Lu B., Fisher L.W., Robey P.G., Shi S. (2003). SHED: Stem Cells from Human Exfoliated Deciduous Teeth. Proc. Natl. Acad. Sci. USA.

[7] Wagner W., Horn P., Castoldi M., Diehlmann A., Bork S., Saffrich R., Benes V., Blake J., Pfister S., Eckstein V. (2008). Replicative Senescence of Mesenchymal Stem Cells: A Continuous and Organized Process. PLoS ONE.

[8] Grezella C., Fernandez-Rebollo E., Franzen J., Ventura Ferreira M.S., Beier F., Wagner W. (2018). Effects of Senolytic Drugs on Human Mesenchymal Stromal Cells. Stem Cell Res. Ther..

[9] López-Otín C., Blasco M.A., Partridge L., Serrano M., Kroemer G. (2013). The Hallmarks of Aging. Cell.

[10] Liu L., Cheung T.H., Charville G.W., Hurgo B.M.C., Leavitt T., Shih J., Brunet A., Rando T.A. (2013). Chromatin Modifications as Determinants of Muscle Stem Cell Quiescence and Chronological Aging. Cell Rep..

[11] Lepperdinger G. (2011). Inflammation and Mesenchymal Stem Cell Aging. Curr. Opin. Immunol..

[12] Pan H., Guan D., Liu X., Li J., Wang L., Wu J., Zhou J., Zhang W., Ren R., Zhang W. (2016). SIRT6 Safeguards Human Mesenchymal Stem Cells from Oxidative Stress by Coactivating NRF2. Cell Res..

[13] Lavasani M., Robinson A.R., Lu A., Song M., Feduska J.M., Ahani B., Tilstra J.S., Feldman C.H., Robbins P.D., Niedernhofer L.J. (2012). Muscle-Derived Stem/Progenitor Cell Dysfunction Limits Healthspan and Lifespan in a Murine Progeria Model. Nat. Commun..

[14] Singh L., Brennan T.A., Kim J.-H., Egan K.P., McMillan E.A., Chen Q., Hankenson K.D., Zhang Y., Emerson S.G., Johnson F.B. (2013). Long-Term Functional Engraftment of Mesenchymal Progenitor Cells in a Mouse Model of Accelerated Aging. Stem Cell. Dayt. Ohio.

[15] Kaplon J., Zheng L., Meissl K., Chaneton B., Selivanov V.A., Mackay G., van der Burg S.H., Verdegaal E.M.E., Cascante M., Shlomi T. (2013). A Key Role for Mitochondrial Gatekeeper Pyruvate Dehydrogenase in Oncogene-Induced Senescence. Nature.

[16] Herranz N., Gil J. (2016). Mitochondria and Senescence: New Actors for an Old Play. EMBO J..

[17] Liu B., Meng Q., Gao X., Sun H., Xu Z., Wang Y., Zhou H. (2023). Lipid and Glucose Metabolism in Senescence. Front. Nutr..

[18] Takubo K., Nagamatsu G., Kobayashi C.I., Nakamura-Ishizu A., Kobayashi H., Ikeda E., Goda N., Rahimi Y., Johnson R.S., Soga T. (2013). Regulation of Glycolysis by Pdk Functions as a Metabolic Checkpoint for Cell Cycle Quiescence in Hematopoietic Stem Cells. Cell Stem Cell.

[19] Simsek T., Kocabas F., Zheng J., DeBerardinis R.J., Mahmoud A.I., Olson E.N., Schneider J.W., Zhang C.C., Sadek H.A. (2010). The Distinct Metabolic Profile of Hematopoietic Stem Cells Reflects Their Location in a Hypoxic Niche. Cell Stem Cell.

[20] Ray P.D., Huang B.-W., Tsuji Y. (2012). Reactive Oxygen Species (ROS) Homeostasis and Redox Regulation in Cellular Signaling. Cell. Signal..

[21] Ahlqvist K.J., Suomalainen A., Hämäläinen R.H. (2015). Stem Cells, Mitochondria and Aging. Biochim. Biophys. Acta BBA-Bioenerg..

[22] Zhao Y., Chen B., Shen J., Wan L., Zhu Y., Yi T., Xiao Z. (2017). The Beneficial Effects of Quercetin, Curcumin, and Resveratrol in Obesity. Oxid. Med. Cell. Longev..

[23] Anand David A.V., Arulmoli R., Parasuraman S. (2016). Overviews of Biological Importance of Quercetin: A Bioactive Flavonoid. Pharmacogn. Rev..

[24] Guo C.-Y., Yang C.-F., Li Q.-L., Tan Q., Xi Y.-W., Liu W.-N., Zhai G.-X. (2012). Development of a Quercetin-Loaded Nanostructured Lipid Carrier Formulation for Topical Delivery. Int. J. Pharm..

[25] Vicentini F.T.M.C., Fonseca Y.M., Pitol D.L., Iyomasa M.M., Bentley M.V.L.B., Fonseca M.J.V. (2010). Evaluation of Protective Effect of a Water-in-Oil Microemulsion Incorporating Quercetin against UVB-Induced Damage in Hairless Mice Skin. J. Pharm. Pharm. Sci..

[26] de Oliveira M.R., Nabavi S.M., Braidy N., Setzer W.N., Ahmed T., Nabavi S.F. (2016). Quercetin and the Mitochondria: A Mechanistic View. Biotechnol. Adv..

[27] Ramanan M., Sinha S., Sudarshan K., Aidhen I.S., Doble M. (2016). Inhibition of the Enzymes in the Leukotriene and Prostaglandin Pathways in Inflammation by 3-Aryl Isocoumarins. Eur. J. Med. Chem..

[28] Sudarshan K., Boda A.K., Dogra S., Bose I., Yadav P.N., Aidhen I.S. (2019). Discovery of an Isocoumarin Analogue That Modulates Neuronal Functions via Neurotrophin Receptor TrkB. Bioorg. Med. Chem. Lett..

[29] Mirza M.A., Mahmood S., Hilles A.R., Ali A., Khan M.Z., Zaidi S.A.A., Iqbal Z., Ge Y. (2023). Quercetin as a Therapeutic Product: Evaluation of Its Pharmacological Action and Clinical Applications—A Review. Pharmaceuticals.

[30] Ivan A., Cristea M.I., Telea A., Oprean C., Galuscan A., Tatu C.A., Paunescu V. (2023). Stem Cells Derived from Human Exfoliated Deciduous Teeth Functional Assessment: Exploring the Changes of Free Fatty Acids Composition during Cultivation. Int. J. Mol. Sci..

[31] Jin X., Su R., Li R., Song L., Chen M., Cheng L., Li Z. (2016). Amelioration of Particulate Matter-Induced Oxidative Damage by Vitamin c and Quercetin in Human Bronchial Epithelial Cells. Chemosphere.

[32] Mutlu A.S., Duffy J., Wang M.C. (2021). Lipid Metabolism and Lipid Signals in Aging and Longevity. Dev. Cell.

[33] Currie E., Schulze A., Zechner R., Walther T.C., Farese R.V. (2013). Cellular Fatty Acid Metabolism and Cancer. Cell Metab..

[34] Leekumjorn S., Cho H.J., Wu Y., Wright N.T., Sum A.K., Chan C. (2009). The Role of Fatty Acid Unsaturation in Minimizing Biophysical Changes on the Structure and Local Effects of Bilayer Membranes. Biochim. Biophys. Acta.

[35] Wang Z.-J., Liang C.-L., Li G.-M., Yu C.-Y., Yin M. (2007). Stearic Acid Protects Primary Cultured Cortical Neurons against Oxidative Stress. Acta Pharmacol. Sin..

[36] Fukaya M., Sato Y., Kondo S., Adachi S.-I., Yoshizawa F., Sato Y. (2021). Quercetin Enhances Fatty Acid β-Oxidation by Inducing Lipophagy in AML12 Hepatocytes. Heliyon.

[37] Hamsanathan S., Gurkar A.U. (2022). Lipids as Regulators of Cellular Senescence. Front. Physiol..

[38] Périchon R., Bourre J.M. (1995). Peroxisomal β-Oxidation Activity and Catalase Activity during Development and Aging in Mouse Liver. Biochimie.

[39] Sampath H., Ntambi J.M. (2005). The Fate and Intermediary Metabolism of Stearic Acid. Lipids.

[40] Park S.-H., Nam B.E., Kim J.G. (2019). Shade and Physical Support Are Necessary for Conserving the Aristolochia Contorta Population. Ecol. Eng..

[41] Bermudez B., Dahl T.B., Medina I., Groeneweg M., Holm S., Montserrat-de la Paz S., Rousch M., Otten J., Herias V., Varela L.M. (2017). Leukocyte Overexpression of Intracellular NAMPT Attenuates Atherosclerosis by Regulating PPARγ-Dependent Monocyte Differentiation and Function. Arterioscler. Thromb. Vasc. Biol..

[42] Wang M., Wang B., Wang S., Lu H., Wu H., Ding M., Ying L., Mao Y., Li Y. (2021). Effect of Quercetin on Lipids Metabolism Through Modulating the Gut Microbial and AMPK/PPAR Signaling Pathway in Broilers. Front. Cell Dev. Biol..

[43] Wang M., Wang B., Zhou S., Liu J., Lu H., Wu H., Ding M., Li Y. (2022). Quercetin Ameliorates Chicken Quality by Activating the PI3K/PKB/AMPK Signaling Pathway in Broilers. Front. Vet. Sci..

[44] Chistiakov D.A., Sobenin I.A., Revin V.V., Orekhov A.N., Bobryshev Y.V. (2014). Mitochondrial Aging and Age-Related Dysfunction of Mitochondria. BioMed Res. Int..

[45] Röhrdanz E., Bittner A., Tran-Thi Q.-H., Kahl R. (2003). The Effect of Quercetin on the mRNA Expression of Different Antioxidant Enzymes in Hepatoma Cells. Arch. Toxicol..

[46] Gao W., Pu L., Chen M., Wei J., Xin Z., Wang Y., Yao Z., Shi T., Guo C. (2017). Glutathione Homeostasis Is Significantly Altered by Quercetin via the Keap1/Nrf2 and MAPK Signaling Pathways in Rats. J. Clin. Biochem. Nutr..

[47] Perez-Vizcaino F., Fraga C.G. (2018). Research Trends in Flavonoids and Health. Arch. Biochem. Biophys..

[48] Brawner K.M., Yeramilli V.A., Duck L.W., Van Der Pol W., Smythies L.E., Morrow C.D., Elson C.O., Martin C.A. (2019). Depletion of Dietary Aryl Hydrocarbon Receptor Ligands Alters Microbiota Composition and Function. Sci. Rep..

[49] Huang J., Wang Y., Zhou Y. (2022). Beneficial Roles of the AhR Ligand FICZ on the Regenerative Potentials of BMSCs and Primed Cartilage Templates. RSC Adv..

[50] Salehi B., Machin L., Monzote L., Sharifi-Rad J., Ezzat S.M., Salem M.A., Merghany R.M., El Mahdy N.M., Kılıç C.S., Sytar O. (2020). Therapeutic Potential of Quercetin: New Insights and Perspectives for Human Health. ACS Omega.

[51] Wu Q.-J., Zhang T.-N., Chen H.-H., Yu X.-F., Lv J.-L., Liu Y.-Y., Liu Y.-S., Zheng G., Zhao J.-Q., Wei Y.-F. (2022). The Sirtuin Family in Health and Disease. Signal Transduct. Target. Ther..

[52] Yoshida K., Kusama K., Shinohara G., Sato S., Yoshie M., Tamura K. (2024). Quercetin Stimulates Trophoblast Fusion via the Mitochondrial Function. Sci. Rep..

[53] Santos-Lozano A., Santamarina A., Pareja-Galeano H., Sanchis-Gomar F., Fiuza-Luces C., Cristi-Montero C., Bernal-Pino A., Lucia A., Garatachea N. (2016). The Genetics of Exceptional Longevity: Insights from Centenarians. Maturitas.

[54] Kabziński J., Walczak A., Mik M., Majsterek I. (2019). Sirt3 Regulates the Level of Mitochondrial DNA Repair Activity through Deacetylation of NEIL1, NEIL2, OGG1, MUTYH, APE1 and LIG3 in Colorectal Cancer. Pol. Przegl. Chir..

[55] Xin T., Lu C. (2020). SirT3 Activates AMPK-Related Mitochondrial Biogenesis and Ameliorates Sepsis-Induced Myocardial Injury. Aging.

[56] Smirnov D., Eremenko E., Stein D., Kaluski S., Jasinska W., Cosentino C., Martinez-Pastor B., Brotman Y., Mostoslavsky R., Khrameeva E. (2023). SIRT6 Is a Key Regulator of Mitochondrial Function in the Brain. Cell Death Dis..

[57] Mei Z., Zhang X., Yi J., Huang J., He J., Tao Y. (2016). Sirtuins in Metabolism, DNA Repair and Cancer. J. Exp. Clin. Cancer Res..

[58] Mao Z., Hine C., Tian X., Van Meter M., Au M., Vaidya A., Seluanov A., Gorbunova V. (2011). SIRT6 Promotes DNA Repair under Stress by Activating PARP1. Science.

[59] Tang M., Tang H., Tu B., Zhu W.-G. (2021). SIRT7: A Sentinel of Genome Stability. Open Biol..

[60] Lagunas-Rangel F.A. (2019). Current Role of Mammalian Sirtuins in DNA Repair. DNA Repair.

