# Supplementary material for: Quercetin and Mesenchymal Stem Cell Metabolism: A Comparative Analysis of Young and Senescent States
Source: Molecules. 2024 Dec 5;29(23):5755. doi: 10.3390/molecules29235755 (PMC11643090; doi:10.3390/molecules29235755)
Supplement: Supplementary file 1 [file molecules-29-05755-s001.zip › molecules-3357341-supplementary.pdf]

Supplementary materials

Quercetin and stem cell metabolism: A comparative analysis of young and senescent states

Alexandra Ivan[1,2], Alexandra Teodora Lukinich-Gruia[2,*], Iustina-Mirabela Cristea[2], Maria-Alexandra Pricop[2,3], Crenguta Livia Calma[1,2], Alina-Georgiana Simina[2], Calin Adrian Tatu[1,2*], Atena Galuscan[4,5], Virgil Paunescu[1,2]

[1]Department of Functional Sciences, Center of Immuno-Physiology (CIFBIOTEH), University of Medicine and Pharmacy "Victor Babes", Eftimie Murgu Sq. 2, Timisoara, 300041, Romania.

[2]OncoGen Centre, Clinical County Hospital "Pius Branzeu", Blvd. Liviu Rebreanu 156, 300723, Timisoara, Romania.

[3]Department of Applied Chemistry and Environmental Engeneering and Inorganic Compounds, Faculty of industrial Chemistry, Biotechnology and Environmental Engeneering, Polytechnic University of Timisoara, Vasile Pârvan 6, 300223, Timisoara, Romania

[4]Translational and Experimental Clinical Research Centre in Oral Health, Department of Preventive, Community Dentistry and Oral Health, "Victor Babes" University of Medicine and Pharmacy, 300040 Timisoara, Romania; galuscan.atena@umft.ro (A.G.)

[5]Department I, Department of Preventive, Community Dentistry and Oral Health, "Victor Babes" University
of Medicine and Pharmacy, Eftimie Murgu Sq. No 2, 300041 Timisoara, Romania

* Correspondence: alexandra.gruia@hosptm.ro (ATLG); geomed88@gmail.com (CAT).

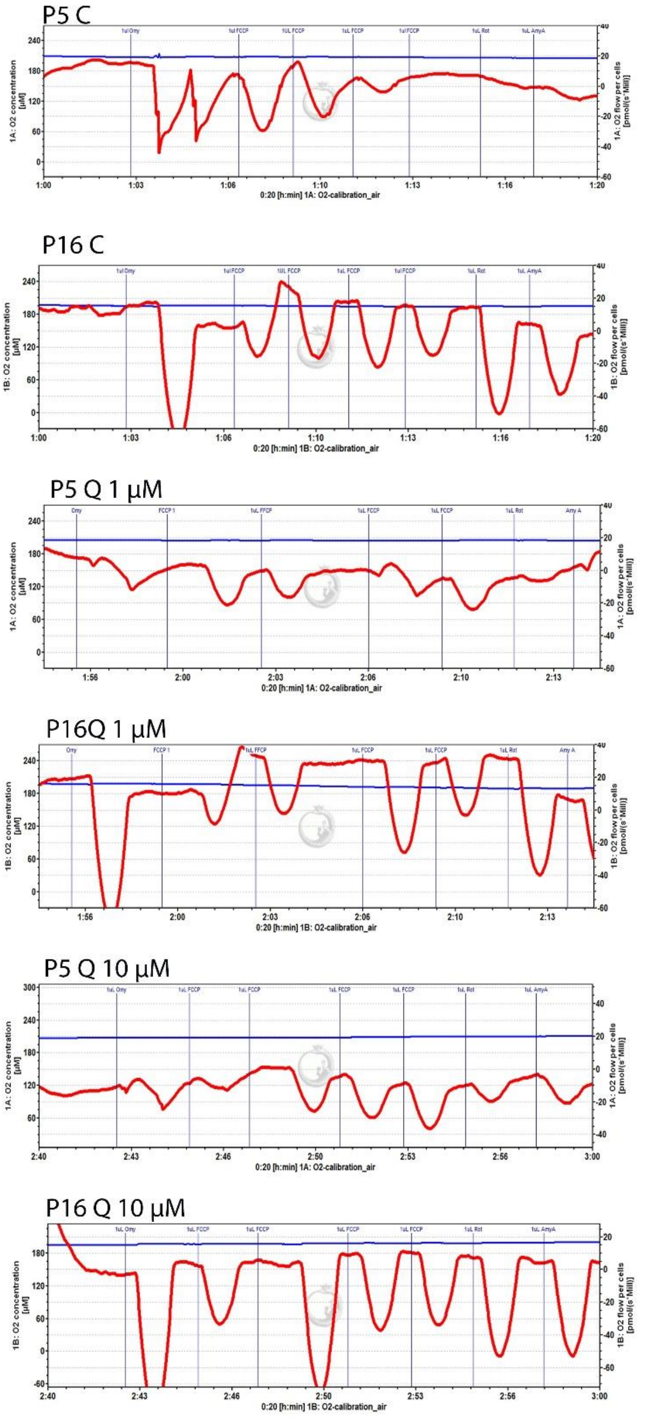

Figure S1. Coupled and Uncoupled Respiration of Intact Cells: Representative Oroboros oxygraphs depicting oxygen consumption of SHED cells showing basal oxygen consumption, measured using high-resolution respirometry. Traces include oligomycin-insensitive respiration (following oligomycin addition) and subsequent responses to sequential additions of FCCP (to assess maximal respiratory capacity), rotenone (Rot), and antimycin A (AmyA) to inhibit specific mitochondrial complexes.

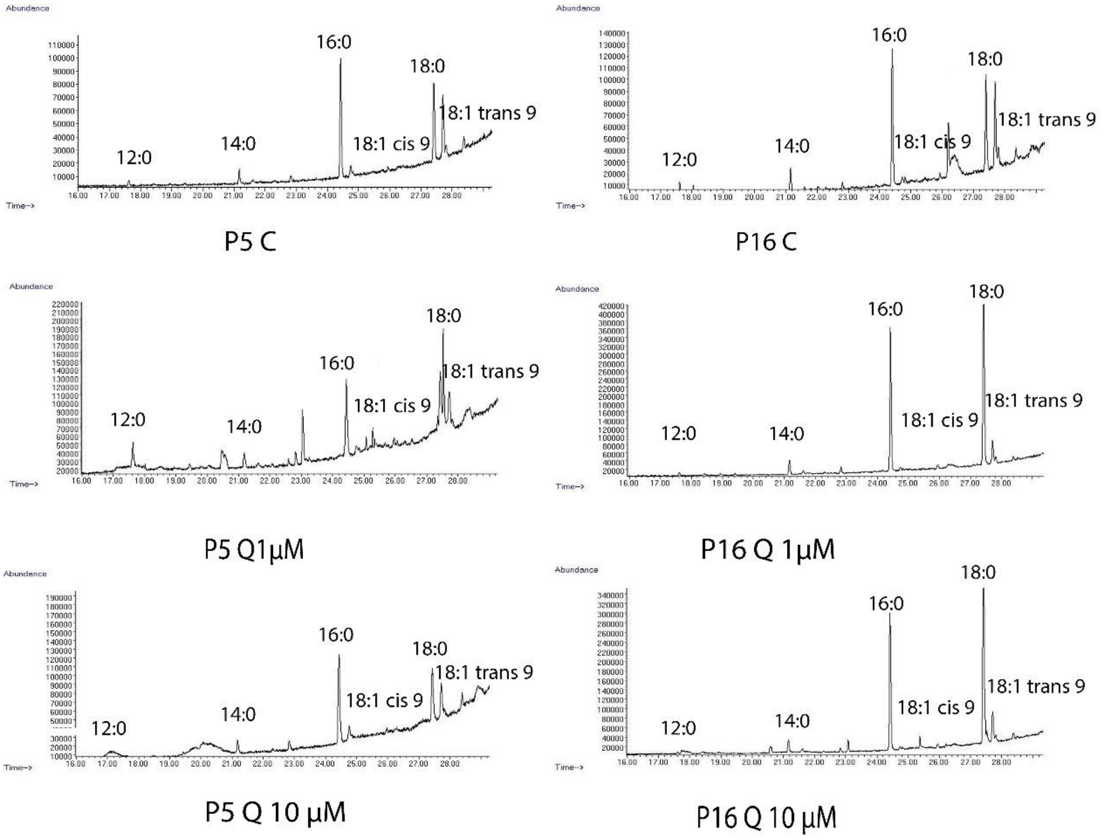

Figure S2. Representative chromatogram detected by GC-MS showing major fatty acids methyl esters (FAME): lauric acid methyl ester (12:0), myristic acid methyl ester (14:0), palmitic acid methyl ester (16:0), stearic acid methyl ester (18:0), oleic acid methyl ester (18:1 cis 9) and elaidic acid methyl ester (18:1 trans 9).

Table S1. Primer sequence for q-PCR analysis

Gene	*Primer sequence*
PPARg	5′-aagaccactcccactcctttg-3′
	5′-gtcagcggactctggattca-3′
ACCa	5′-tcacacctgaagaccttaaagcc-3′
	5′-agcccacactgcttgtactg-3′
	5′-agcccacactgcttgtactg-3′
Ahr 3-5	5′-tctatgccgcttggaaggat-3′
	5′-acatcacctacgccagtgc-3′
SOD	5′-ggtgggccaaaggatgaagag-3′
	5′-ccacaagccaaacgacttcc-3′
CYP1A1	5′-tcggccacggagtttcttc-3′
	5′-ggtcagcatgtgcccaatca-3′
SIRT 1	5′-tagccttgtcagataaggaagga-3′
	5′-tgttctgggtatagttgcaagt-3′
SIRT 3	5′-acccagtggcattccagac-3′
	5′-ggcttggggttgtgaaagaag-3′
SIRT4	5′-aagctgagctttgcgttgact-3′
	5′-ccaatggaggctttcgagca-3′
SIRT 5	5′-tccagcgtccacacgaaa-3′
	5′-aacttggccgagccattttca-3′
SIRT6	5′-cccacggagtctggaccat-3′
	5′-ctctgccagtttgtccctg-3′
SIRT7	5′-gacctggtaacggagctgc-3′
	5′-cgaccaagtatttggcgttcc-3′

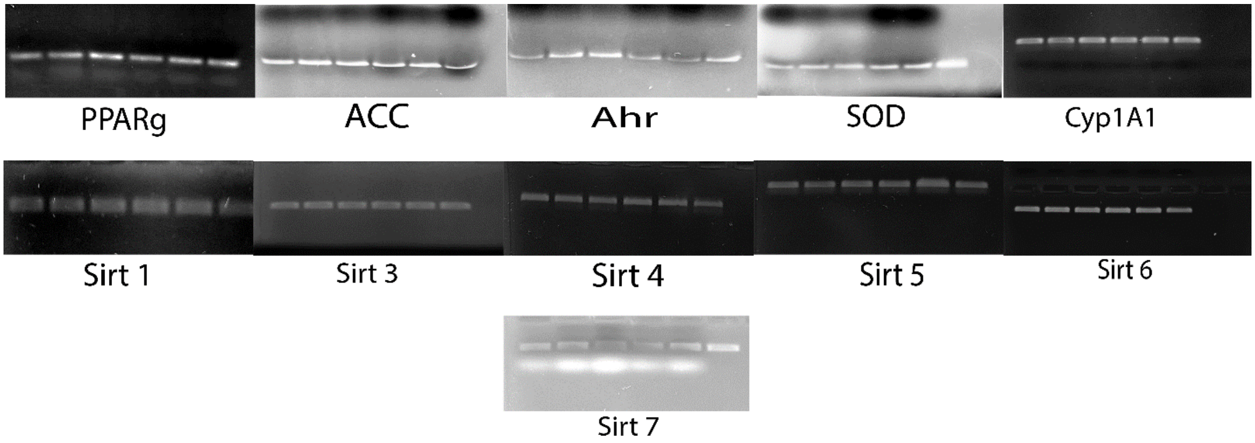

Figure S3. Agarose gel electrophoresis of qPCR products for oxidative stress-related genes and sirtuins.
